# The medical relevance of *Spirometra* tapeworm infection in Indonesian Bronzeback snakes (*Dendrelaphis pictus*): A neglected zoonotic disease

**DOI:** 10.14202/vetworld.2019.844-848

**Published:** 2019-06-18

**Authors:** Aditya Yudhana, Ratih Novita Praja, Arif Supriyanto

**Affiliations:** 1 Department of Parasitology, Faculty of Veterinary Medicine, Airlangga University, Indonesia; 2 Department of Microbiology, Faculty of Veterinary Medicine, Airlangga University, Indonesia; 3 Department of Wildlife Conservation, Education Staff of Mojokerto Reptile and Exotic Animals Community, Indonesia

**Keywords:** *Dendrelaphis pictus*, sparganosis, *Spirometra*, zoonosis

## Abstract

**Aim:**

*Spirometra* parasites cause sparganosis, a zoonotic disease, especially in reptiles and humans. This study aimed to report on the prevalence and effects of *Spirometra* parasites infection on public health and provide a scientific foundation for its prevention.

**Materials and Methods:**

A total of 378 living Indonesian wild-caught and captive-bred Bronzeback snakes (*Dendrelaphis pictus*) were selected. The snakes were euthanized using ethyl ether anesthesia before checking for *Spirometra* parasites. The numbers of *Spirometra* located in the muscle tissue, subcutaneous tissue, and coelom (including the viscera) were each counted to investigate the distribution of *Spirometra* inside the snake body cavity.

**Results:**

The total prevalence in the sample was 50.85%. The prevalence values in wild-caught and captive-bred snakes were 70.7% and 48.7%, respectively. More than half (56.6%) of the *Spirometra* parasites were located in the muscular tissue, while 29.5% were in the subcutaneous tissue and 13.8% were in the coelomic cavity.

**Conclusion:**

Wild-caught Indonesian Bronzeback snakes, which are sold as food in markets, and captive-bred snakes, which are collected as exotic pets in Indonesia, have similar opportunities to transmit the *Spirometra* parasite and cause global health problems due to their high prevalence.

## Introduction

In current times, reptiles are threatened by factors influencing extinction, such as habitat loss, environmental pollution, infectious diseases, unexpected uses, and global climate change [1]. One of these endangered reptiles is the snake. Snakes are one of the several types of reptiles whose existence is protected by government law. Snakes are also included in the group of exotic animals that have been hunted to be sold or domesticated. This animal plays an important role in maintaining ecosystem stability [2]. *Dendrelaphis pictus*, more commonly known as the Indonesian Bronzeback snake, has a wide distribution, extending from the Western to Eastern regions of Indonesia with origins in the province of East Java. Nowadays, the Indonesian Bronzeback snakes from East Java are included in snake species who face extinction [3]. Various environmental threats have not only reduced the snake population but also increased health problems such as parasitic diseases from tapeworm infections [4]. Sparganosis is a parasitic disease in snakes caused by the infective stage of *Spirometra* tapeworms; it is a zoonotic disease, which means it can be transmitted to humans and cause clinical signs such as allergic reaction, chronic inflammation, and painful nodules within subcutaneous tissues [5].

Sparganosis is an infection of humans and animals caused by plerocercoid larvae (spargana) originating from various diphyllobothroid tapeworms belonging to the genus *Spirometra* [6]. Spargana live in frogs and snakes who serve as the second intermediate hosts of *Spirometra*, but they can also infect humans, pigs, rodents, and birds who serve as other intermediate or paratenic hosts. Carnivores, such as dogs and cats, serve as the final hosts of *Spirometra*, which parasitize in their small intestines [7]. Moreover, parasitic disease in wildlife may have a significant influence on domestic animals and humans. One of the possible sources of infection is the consumption of meat of wild animals, such as snakes that enable circulation of infectious agents in the environment and their transmission to humans and domesticated animal hosts. *Spirometra* tapeworm has become one of the most neglected parasitic agents with zoonotic potential [8].

To the best of our knowledge, there have been no studies about *Spirometra* parasite infection in Indonesian Bronzeback snakes. Therefore, we aimed to report about the prevalence and public health effects of *Spirometra* infection and provides a scientific foundation for preventing sparganosis, to further our understanding of its medical relevance.

## Materials and Methods

### Ethical approval

This study was conducted with permission from the local reptile community and wildlife conservation department in East Java Province, Indonesia. This study was reviewed and approved by the Animal Care and Use Committee of Faculty of Veterinary Medicine, Universitas Airlangga, number 713-KE.

### Sampling

A total of 378 living Indonesian wild-caught and captive-bred Bronzeback snakes were selected from the reptile market in Mojokerto City, East Java Province, Indonesia (112.434084 Longitude and −7.472638 Latitude). Snake species were identified according to their morphological characteristics. *D. pictus* snakes were characterized by their head color ranging from brown to bronze at the top; further, a black eye-stripe extends along the neck for a short distance down the body. In addition, there was a cream and black stripe along the flanks. The larger vertebral scales, which run along the full length of the body, may be brown or olive gray. When threatened or when consuming prey, this snake inflates its body slightly to reveal bluish or turquoise skin underlying its body scales. The head was slightly larger than its moderately slender body, and its eyes were large, typically with a brown iris. The snakes were kept in the Reptile Rescue Center by the local reptile community management. We conducted this work with permission from the local reptile community and wildlife conservation department. The live snakes were euthanized using ethyl ether anesthesia before checking for *Spirometra* parasites. *Spirometra* was identified by its tape-like morphology with a white colored, long, flat, and segmented body form.

The snakes were dissected to examine for infection from *Spirometra* parasites. Their body length and weight were measured before the dissection. For each snake, the body was peeled from the neck to the top of the tail, and then, we isolated the visceral masses from the esophagus and trachea to the cloaca. Then, the numbers of *Spirometra* located in the muscle tissue, subcutaneous tissue, and coelom (including viscera) were each counted to investigate the distribution of *Spirometra* inside the snake body.

### Statistical analysis

The data were processed using Software SPSS v21 (IBM, USA). In addition, the non-parametric Kruskal–Wallis test was used to compare the difference in the numbers of parasites among the muscle tissues, subcutaneous tissues, and coeloms of the snakes.

## Results

**Figure-1:**
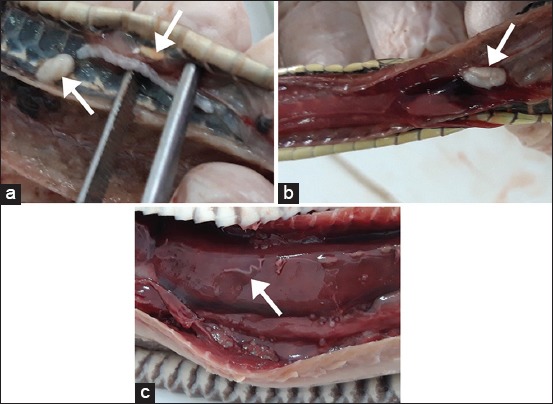
*Spirometra* located in (a) subcutaneous tissue, (b) coelom, and (c) muscle of *Dendrelaphis pictus*. Arrows point to *Spirometra*.

The body length of the Indonesian Bronzeback snakes ranged from 13 to 132 cm and the body weight ranged from 2.1 to 1550.0 g. Essential information, including source; number of samples; and age, body length, and weight of each snake, is shown in Table-1. Overall, *Spirometra* tapeworms were isolated from 378 snakes, and the total prevalence was 50.85% in the total examined snake samples. The prevalence of wild-caught snakes and captive-bred snakes was 70.7% and 48.7%, respectively (Table-2). The macroscopic morphology of *Spirometra*, which infects different tissues, is shown in Figure-1.

**Table-1:** Essential information about the 378 wild-caught and captive-bred snake samples from reptile markets in Mojokerto City, East Java Province, Indonesia.

Species of snakes	Source of samples	Number of samples	Range of body length (cm)	Medians of body length (cm)	Range of body weight (g)	Medians of body weight (g)
***Dendrelaphis pictus***						
*Wild-caught (N=181)						
Baby (1-3 months)	Mojokerto	26	13-32	28	2.1-5.6	3.6
Juvenile (3-6 months)	Mojokerto	83	42-84	73	150.5-403.6	327.8
Adult (6-9 months)	Mojokerto	72	96-110	105	564.8-1301	1105.1
*Captive breed (N=197)						
Baby (1-3 months)	Mojokerto	20	18-52	48	5.5-10.6	8.3
Juvenile (3-6 months)	Mojokerto	91	72-96	83	607.5-836.6	726.8
Adult (6-9 months)	Mojokerto	86	113-132	125	957.8-1550	1458.9
**Total samples**		**378**				

**Table-2:** Prevalence, intensity, and locations of *Spirometra* infections found in wild-caught and captive-bred snake samples from reptile markets in Mojokerto City, East Java Province, Indonesia.

Species of snakes	Infection of *Spirometra*			Locations of *Spirometra*		
		
Wild-caught and captive breed snakes	Prevalence (%)	Intensity of infection	Mean intensity of infection	Subcutaneous tissue	Coelom	Muscle
***Dendrelaphis pictus***						
*Wild-caught (N=181)						
Baby (1-3 months)	34.6	0-23	8.2	7	3	14
Juvenile (3-6 months)	92.7	0-111	14.3	213	154	458
Adult (6-9 months)	58.3	0-87	11.8	198	56	317
*Captive breed (N=197)						
Baby (1-3 months)	15.0	0-7	0.3	2	0	4
Juvenile (3-6 months)	61.5	0-68	13.3	63	31	179
Adult (6-9 months)	43.0	0-43	9.6	89	24	124
**Total**				572	268	1096
**Mean**	**50.85**	**0-56.5**	9.6	3	2	7

A Kruskal–Wallis test showed a significant difference among the number of *Spirometra* infections in the subcutaneous tissue, coelom, and muscle (p<0.05).

**Figure-2:**
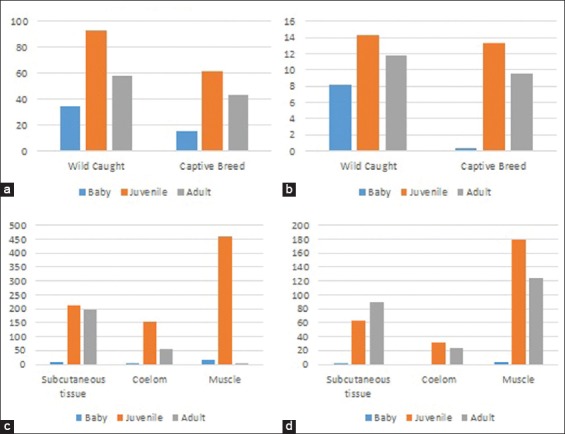
(a) Prevalence of *Spirometra*, (b) intensity of *Spirometra* infection, (c) number of *Spirometra* found in wild-caught snakes, and (d) number of *Spirometra* found in captive-bred snakes.

More than half (56.6%) of the *Spirometra* parasites were located in muscular tissue of the snakes, while 29.5% were in the subcutaneous tissue and 13.8% were in the coelomic cavity (Figure-2). The non-parametric test showed that the density distributions among the muscle tissue, subcutaneous tissue, and coelom were significantly different (p<0.05) (Table-2, Figure-2). The prevalence and infection intensities of *Spirometra* were different according to snake age and source (Figure-2). Wild-caught snakes had higher prevalence and infection intensities compared to captive-bred snakes. Wild-caught snakes in the juvenile age group had the highest prevalence (92.7%) compared to 34.6% in the baby age group and 58.3% in the adult age group. Captive-bred snakes also recorded a higher prevalence (61.5%) in the juvenile age group compared to 15% in the baby age group and 43% in the adult age group.

## Discussion

Indonesian Bronzeback snakes have not only become popular as exotic pets in Indonesia but also as raw food for human consumption. Indonesian Bronzeback snakes collected in this study belong to the Colubridae family and are mostly sold in reptile or food markets throughout Asia [9]. Spargana or larvae of *Spirometra* found in this study have a fairly high prevalence (50.85%) in the wild-caught and captive-bred Indonesian Bronzeback snakes collected as samples in this study, which indicates that more than half of the snakes are infected by spargana. Similar results were obtained in studies in other areas and other species of snakes [10]. The type of diet commonly consumed by snakes will also usually affect the prevalence of *Spirometra* infection. Wild-caught snakes are usually infected from consuming frogs as a food source. Due to this, the wild-caught snakes in food markets in China are generally infected by spargana, which spreads to different tissues such as subcutaneous tissues, the coelom, and muscles of snakes [11]. Spargana infection was also reported in two Russell viper snakes from Chennai Snake Park Trust, India, during a gross examination method revealing spargana in the subcutaneous tissue of the snakes [12]. Due to the high prevalence of infection in snakes, snake consumption poses a great risk for human sparganosis because the spargana not only cause parasitic diseases in snakes but also in humans.

Throughout Asia, several cases of sparganosis have been reported in humans and reptiles. Sparganosis in reptiles, including snakes, has also been globally reported, with a high prevalence in several Asian countries, such as South Korea, Japan, Thailand, and China [13]. The occurrence of snakes as the main source of human sparganosis in Korea was due to consumption of snake meat for daily food [14]. In China, there are many cases of human sparganosis caused by eating raw meat from snakes and frogs, drinking snake blood, and swallowing snake gall bladders. Using improper cooking methods for snakes also increase the risk of infection. In addition, *Spirometra* may contaminate food during the process of cooking snake meat. In a recent report from 2011, a human patient suffered from bronchial sparganosis because he had a history of ingesting raw frogs, snakes, and drinking raw snake blood [15]. Another case of cerebral sparganosis was reported the next year, in 2012, from eating frogs and snakes [16]. Another recent report showed 104 cases of human sparganosis from 2000 to 2006, and more than half, or 53.9%, of cases were caused by eating snakes or frogs [17].

When humans are infected by *Spirometra* larvae, commonly known as spargana, the larvae can perform visceral migration, infect many tissues, and develop into the mature stage. Subcutaneous sparganosis is the most common form among sparganosis in humans. Spargana has also been reported to migrate into subcutaneous tissues and peripheral muscles such as abdominal walls, lower extremities, scrotums, and chest walls. Under the skin, the lesions look like rubbery and irregular lumps or nodules of 1-2 cm long that resemble a lipoma or fibroma, while causing itchiness, inflammation, and pain. Some infected patients have had chronic forms, and sometimes, the nodules can switch from one tissue to another [18]. Consumption of raw meat or any half-cooked snake biological products can increase the risk of sparganosis. The larvae of *Spirometra* are very soft and thin, and people generally do not closely examine them, so they conclude that snake meat that is prepared as food is in a condition of proper hygiene and is safe to serve as a food source. From this, snake meat is suspected of playing a role in sparganosis transmission, which is related to human sparganosis.

In this study, the prevalence and intensity of *Spirometra* infection have been different among various snake age groups. We found interesting data that *D. pictus* of juvenile age have the highest prevalence and intensity of infection compared to baby and adult age groups. Theoretically, snakes in the baby age group are more susceptible to parasite infections. However, juvenile snakes may have larger and longer bodies, so they also have wider range to hunt the prey, on mainly frogs, which play a role as the intermediate host of *Spirometra*. Juvenile snakes also have immunity against infection, but they are not as stable as adult snakes, which more frequently adapt to infectious diseases in wild environments. As a result, we infer that snakes in the juvenile age group may have a higher risk of infection to *Spirometra*, and it would be beneficial to conduct further studies on this topic.

There seems to be a relation between *Spirometra* infection and the feeding habits of snakes. In general, snakes in wild environment prefer to prey on frogs as a food source and these habits may lead to *Spirometra* infection [7]. Our investigation is also in accordance with previous studies because wild-caught snakes have higher prevalence and intensity of infection compared to captive-bred snakes. Wild-caught snakes are usually collected and sold in markets as traditional food sources for humans. However, consuming snake meat as a traditional habit occurs in multiple Asian countries, which is related to numerous sparganosis cases reported in China [19]. A previous study reported that snakes and other wild-caught animals have resulted in numerous cases of human sparganosis [17]. Therefore, it is necessary to strengthen food safety and food security policies so that the transmission of human sparganosis from wild animals, such as snakes, can be prevented early on and the risk of infection can be minimized. Moreover, to the best of our knowledge, there is no data or report about *Spirometra* infection in *D. pictus*, as well as, there is no report about the spread and prevalence of the infection in Indonesia. To further understand the causes of *Spirometra* infection in snakes, more detailed surveys on the habitats and dietary habit of these snakes are necessary.

## Conclusion

In this present study about *Spirometra* infection in *D. pictus*, we indicated that wild-caught snakes, which are sold as food in markets, and captive-bred snakes, which are collected as exotic pets in Indonesia, have similar opportunities for *Spirometra* infection and both cause public health problems due to their high infection prevalence. Based on this present study, we also suggest some prevention measures for sparganosis in human and animals such as publishing scientific data about the epidemiology and harm of sparganosis and applying government and local laws to strengthen and effectively control and monitor the illegal trade of wild-caught snakes in food markets. Moreover, further studies on the occurrence of sparganosis in wildlife and *Spirometra* identification through molecular avenues can be conducted to globally complete the prevalence data and increase the public awareness of a neglected zoonotic disease.

## Authors’ Contributions

AY is a supervised and project leader. AY is a data analysis and collected samples and RNP carried out dissection of snake samples. AS carried out the collection of snake samples. All authors contributed to the drafting and revision of the manuscript. All authors read and approved the final manuscript.
